# Fast and robust multiple sequence alignment with phylogeny-aware gap placement

**DOI:** 10.1186/1471-2105-13-129

**Published:** 2012-06-13

**Authors:** Adam M Szalkowski

**Affiliations:** 1 Department of Computer Science, ETH Zürich, Universitätstrasse, Switzerland; 2 Swiss Institute of Bioinformatics, Switzerland

## Abstract

**Background:**

ProGraphMSA is a state-of-the-art multiple sequence alignment tool which produces phylogenetically sensible gap patterns while maintaining robustness by allowing alternative splicings and errors in the branching pattern of the guide tree.

**Results:**

This is achieved by incorporating a graph-based sequence representation combined with the advantages of the phylogeny-aware gap placement algorithm of Prank. Further, we account for variations in the substitution pattern by implementing context-specific profiles as in CS-Blast and by estimating amino acid frequencies from input data.

**Conclusions:**

ProGraphMSA shows good performance and competitive execution times in various benchmarks.

## Background

Multiple sequence alignment (MSA) is often the first step for evolutionary analyses of protein families. Its role is to detect homologous characters and to reconstruct the evolutionary history relating a set of sequences.

ProGraphMSA combines the advantages of several state-of-the-art methods [1] with an efficient implementation to provide fast and accurate multiple sequence alignments. This tool includes methods like progressive partial order alignment [2] combined with phylogeny-aware gap placement [3], which causes the gaps in the multiple sequence alignment to principally follow the branching pattern of the guide tree, but still allows for exceptions to account for alternative splicings and errors in the guide tree. This work was motivated by discussions with Dr. Löytynoja, the author of Prank who is also working on a graph-alignment algorithm combined with phylogeny-aware gap placement [4] with a focus on the placement of sequenced data onto a reference alignment/sequence.

To account for the uncertainty in pair-wise distance estimates a BioNJ [5] guide tree is used. ProGraphMSA achieves competitive execution times thanks to alignment-free distances [6] for constructing an approximate initial guide tree.

As evolution is not uniform along a sequence, a site-independent Markov model is often not able to account for specific substitution patterns and evolutionary rates in e.g. secondary structure elements, low complexity regions, or intrinsically disordered proteins. Several specific substitution matrices have been proposed for these structures [7,8], which could be combined into e.g. a Hidden Markov Model (HMM), but adding states to the alignment HMM would significantly affect the execution time.

Instead, we implement *context-specific profiles*[9] which directly infer the substitution pattern of a site from the site's context. The method uses a library containing 4000 context profiles covering a large spectrum of possible evolutionary scenarios. To our knowledge, ProGraphMSA is the first software to apply context-specific profiles to the alignment of multiple sequences and thereby significantly increasing alignment accuracy.

## Implementation

ProGraphMSA is based on progressive alignment as this has the advantage of having a linear time complexity with respect to the number of sequences and implies phylogenetically sensible evolutionary events. Unfortunately, this can also be a disadvantage, as errors in the guide tree or unexpected events such as alternative splicings might induce errors in the alignment. A graph-based sequence representation is able to store the whole history of indel events and thus allows for handling these cases by reverting an indel introduced by an earlier step of the progressive alignment.

### Graph-based alignment

**Figure 1:**
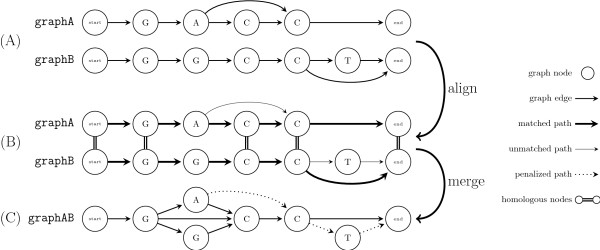
**Sequence graph.** graphA and graphB, each containing an alternative path due to indels in a previous alignment (**A**), are aligned with a variant of the Needleman-Wunsch algorithm [10] (**B**), which selects homologous paths during backtracking (thick arrows). Double lines indicate matched nodes (start and end nodes are always matched). In our example the selected homologous paths indicate that the additional edge in graphA is a deletion and the "T" in graphB is an insertion. For new indels (unmatched nodes along the homologous path) new edges are added to the graph representing the ambiguity, that these new indels might be either insertions or deletions. Finally, the graphs are merged according to the homologies defined by the alignment (**C**). The paths through graphsAB represent the possible ancestral sequences in which unused paths (dotted) are annotated with a penalty score and are less likely to be reused in subsequent alignments.

Ancestral sequences at internal tree nodes are represented as directed acyclic graphs [2] with explicit start and end nodes (Figure 1). All internal nodes correspond to sequence characters and the edges are used to track the indel history in the alignment along the corresponding sub-tree. Every path through the graph can be interpreted as a possible ancestral sequence.

The knowledge of all past indel events prevents the repeated penalization of insertions and alternative splicings [2]. Further, the graph-based representation is able to attenuate a weak point of progressive alignment. This allows for wrongly inferred indels to be revoked [4] rendering the algorithm more robust against small errors in the guide tree.

At each step of the progressive alignment two graphs are aligned using a variant of the Needleman-Wunsch algorithm [10] with affine gap penalties [11]. These algorithms are instances of the Viterbi dynamic programming algorithm [12] and are originally designed for the alignment of sequences. The alignment score in each cell of a dynamic programming matrix is computed as the maximum of possible transitions from three adjacent cells in diagonal, horizontal or vertical direction. These transitions correspond to either matching two homologous characters of the sequences or a gap in one of the sequences.

The leaves of the guide tree are assigned linear graphs corresponding to the input sequences where every graph node but the start node has exactly one predecessor. In general, the inner nodes of the guide tree can contain arbitrary directed acyclic graphs where graph nodes can have multiple predecessor nodes. Thus for graphs the alignment algorithm has to be extended to consider all combinations of preceding graph nodes for each cell in the dynamic programming matrix. While the alignment of sequences considers three preceding cells, the alignment of graphs has to consider n*m + *n* + *m*preceding cells, if the corresponding graph nodes have *n* and *m* preceding nodes, respectively. This is *n* + *m* for the diagonal direction, when matching two nodes, and *n* or *m* for horizontal or vertical gaps.

Analogous to sequence alignment, the alignment algorithm identifies a homologous path in each graph by backtracking in the dynamic programming matrix. New gaps are created for unmatched nodes along the homologous paths in both graphs but are not immediately distinguished into insertions and deletions. Instead, for each indel two alternative paths are added to the ancestral graph and the decision is postponed. In the original phylogeny-aware gap placement this procedure corresponds to flagging unresolved gap positions in the ancestral sequence [3].

Unlike e.g. in Ortheus [13], the distinction between insertions and deletions is not optimized over all ancestral sequences. Instead the decision is made with the help of the closest outgroup i.e. in the alignment at the next guide tree node towards the root of the tree. Whichever of the alternative paths is aligned to the outgroup graph is considered part of the ancestral sequence (Figure 1). Thus, aligned paths are considered deletions and unmatched paths are considered insertions.

In principle we implement the progressive partial order alignment algorithm [14] augmented with edge weights. These are used to realize a "relaxed" variant of phylogeny-aware gap placement by penalizing paths, which are believed not to be part of the ancestral sequence [4]. Thus, unmatched paths in a graph are penalized with a cost proportional to the evolutionary distance separating the current internal tree node from the last use or the introduction of the path. This corresponds to an exponentially declining probability of the insertion/deletion having been inferred incorrectly. Therefore all indels from previous alignments can be reused, however with an increasing penalty if they have not been matched in a recent alignment.

### Model of evolution

**Figure 2:**
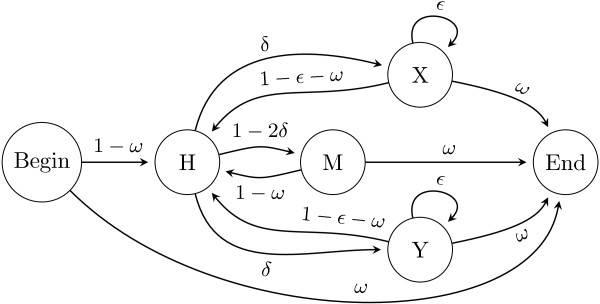
**Pair-HMM for the aligment of two graphs.** Pair-HMM for the alignment of two graphs. *M* is the state emitting aligned node pairs, *X* and *Y * emit a node in one sequence and a gap in the other one, and *H* is a silent transient state. As each graph node is associated with an alignment column, the emission probability of a node or a pair of nodes is equal to the likelihood of the corresponding phylogenetic sub-tree based on the characters of these columns. *δ * is the gap opening probability, *ε* is the gap extension probability, and *ω * is the probability of changing to the terminal state.

Unlike in the progressive partial order alignment algorithm [14], we model the evolution of indels using a pair-HMM (Figure 2), which is used at each internal node of the guide tree for the alignment of the graphs representing the ancestral sequences of the left and right sub-trees. The states *X* and *Y* correspond to gaps with affine penalties in the corresponding graph, *M* is a state matching two homologous graph nodes, and *H* is a silent transient state. The default parameters of the alignment pair-HMM were estimated on BAliBASE version 3.0 [15] or taken from the pair-wise alignment implementation in Darwin [16,17]. These estimated parameters include gap opening rate *δ*, gap extension probability *ε*, and a terminal gap probability *α*. The latter special parameter has been introduced due to the observation that insertions and deletions occur more frequently at the terminal regions of proteins [18] or that often different criteria are used to determine the ends of the sequence (e.g. domain boundaries). This can be achieved without the introduction of additional states in the pair-HMM and thus not increasing the execution time by adjusting the transition scores from/to the HMM start/end states.

The pair-HMM is then transformed into a set of recurrence equations for dynamic programming [19] (p. 85). In general (excluding the start and end nodes) the following equations are used for the computation of the four scores *HMXY* in a dynamic programming cell corresponding to the alignment of nodes *i* and *j*, where Pred(*i*) denotes the predecessor nodes of node *i*.

(1) $$\begin{array}{ll} M(i,j) & =\underset{\left(i^{\prime },j^{\prime })\in \text{Pred}(i)\times \text{Pred}(j\right)}{\max}H(i^{\prime },j^{\prime })+\text{match\_init}\\ & \;+S(i,j)+E(i,i^{\prime })+E(j,j^{\prime }) \end{array}$$

(2) $$X(i,j)=\underset{j^{\prime }\in \text{Pred}(j)}{\max}\left\{\begin{array}{l} H(i,j^{\prime })+\text{gap\_init}+E(j,j^{\prime })\\ X(i,j^{\prime })+\text{gap\_ext}+E(j,j^{\prime }) \end{array}\right)$$

(3) $$Y(i,j)=\underset{i^{\prime }\in \text{Pred}(i)}{\max}\left\{\begin{array}{l} H(i^{\prime },j)+\text{gap\_init}+E(i,i^{\prime })\\ Y(i^{\prime },j)+\text{gap\_ext}+E(i,i^{\prime }) \end{array}\right)$$

(4) $$H(i,j)=\max\left\{\begin{array}{l} M(i,j)\\ X(i,j)\\ Y(i,j) \end{array}\right)$$

Here, match_init, gap_init, and gap_ext are computed from the transition probabilities in the pair-HMM, depending on the specific evolutionary distance separating the aligned graphs as defined by the guide tree [3,16,17]. *E* is a matrix with edge penalties and *S* is a pre-computed matrix of match scores for each pair of graph nodes computed using probabilistic ancestral sequences.

#### Probabilistic ancestral sequences

We define the emission probabilities of MSA columns in the pair-HMMs match and gap states as the likelihood of a sub-tree based on the column's characters at the leaves [20]. For the substitution model we use either the GONNET matrix [21] or WAG [22] with an option to estimate amino acid frequencies from input data ("WAG+F"). This likelihood is computed using Felsenstein's tree-pruning algorithm [23]. Therefore for each MSA column *C* and each possible ancestral character *x* we store the conditional likelihood of the tree *t* based on this column, given that the ancestral character is known to be *x*:

ℒ(t,root=x;C)

For the amino acid alphabet *A* we need to store 20 likelihood values in each graph node. For inner guide tree nodes likelihood values are computed recursively from the partial likelihood values of the left and right sub-trees:

$$\begin{array}{ll} ℒ(t,\text{root}=x;C) & =\underset{y_{L}\in A}{\sum }P_{d_{L}}(y_{L}|x)ℒ\left(t,{\text{root}}_{L}=y_{L};C\right)\\ & \times \underset{y_{R}\in A}{\sum }P_{d_{R}}(y_{R}|x)ℒ(t,{\text{root}}_{R}=y_{R};C), \end{array}$$

where *P*_*d*_(*y*|*x*) is the conditional mutation probability from *x* to *y* at evolutionary distance *d*. For leaf nodes with corresponding sequence character *y* this likelihood is $ℒ(t,\text{root}=x;C)=\delta_{\text{xy}}$^a^. Let *Π*_*x*_ be the equilibrium probability of character *x*, then the total likelihood of the tree based on the column *C* can be computed as:

$$ℒ(t;C)=\underset{x\in A}{\sum }\Pi_{x}ℒ(t,\text{root}=x;C)$$

### Guide tree estimation

Profiling Prank [3] showed that most of its execution time is spent during the all-against-all alignment for the estimation of distances for the initial guide tree. Similar to Muscle [24] we overcome this limitation by using alignment-free distances [6] and simple estimates of variances for the initial BioNJ [5] guide tree. These distances and variances are re-estimated by maximum-likelihood from the resulting MSA using the induced pair-wise alignments. This estimation of distances, guide tree, and alignment is iterated until convergence or until a maximum number of iterations is reached. For typical problem sizes this procedure is still much faster than an all-against-all alignment.

### Context-Specific profiles

Context-specific profiles are a method to generate position-specific substitution matrices from a sequence [9]. The method is based on the assumption that the substitution pattern of a site may depend on the neighbouring sites. Originally, the computation and the alignment of context-specific profiles has been applied to pair-wise sequence alignment and homology search, effecting in increased sensitivity especially for distant homologs. In the following we will briefly describe the original algorithm [9] to compute a context-sensitive profile from a sequence and our adaption of this algorithm for the alignment of multiple sequences.

For each position in the sequence the surrounding sequence window is matched against all profiles in the context profile library. This context profile library was built from a large set of alignments and represents typical profile windows observed in alignments of homologous sequences. The default profile library ("K4000.lib") distributed with CS-Blast [9] consists of 4000 profiles with a width of 13 columns. For a given sequence window (*x*_*i*−7_*..x*_*i* + 7_)=*X*_*i*_ around the *i*-th position of the sequence, the probability of matching profile *p*_*k*_ is computed by

(5) $$P(p_{k}|X_{i})\propto P(p_{k})\overset{7}{\underset{j=-7}{\prod }}p_{k}{\left(j,x_{i+j}\right)}^{w_{j}}.$$

This is the probability of the characters in the sequence window *x*_*i* + *j*_ being emitted by profile column *p*_*k*_(*j*,»). This product is multiplied with the prior *P*(*p*_*k*_) of the profile. As the match probability is to be representative for the center column *x*_*i*_ of the sequence window *X*_*i*_, this product is weighted by *w*_*j*_ according to the declining importance of a site with increasing distance to the center column. As suggested by the authors we use $w_{j}=1.3*0.9^{\left|j\right|}$[9].

The expected probability of the center character *x*_*i*_, mutating to residue *y* is given by

(6) $$P(y|X_{i})\propto \overset{K}{\underset{k=1}{\sum }}p_{k}(0,y)P(p_{k}|X_{i}),$$

i.e. the mutation probabilities are a weighted average of the center columns (*p*_*k*_(0,»)) of all profiles in the profile library. A context-specific profile is obtained by applying equation 6 to each position of a sequence.

ProGraphMSA adopts this method and computes context-specific profiles for the input sequences which are placed at the leaves of the guide tree. In this way the expected context-specific evolution along the terminal branches is encoded in the leaf sequences. However, ProGraphMSA's scoring function relies on probabilistic ancestral sequences. Using Bayes' theorem, context-sensitive profiles can be converted into probabilistic ancestral sequences: $ℒ(t,\text{root}=y;x_{i})\propto \frac{P(y|X_{i})\Pi_{x_{i}}}{\Pi_{y}}$. Again, $\Pi_{x_{i}}$ and *Π*_*y*_ denote the equilibrium amino acid frequencies.

Alignments at internal tree nodes are computed using these probabilistic ancestral sequences at the leaves with the exception that terminal branch lengths are ignored (=0) with respect to the substitution model as the expected evolution along those branches is already encoded in the terminal probabilistic ancestral sequences.

#### Adjusting expected divergence in context-specific profiles

The original algorithm [9] allows for the adjustment of expected sequence divergence in context-specific profiles via the parameter *τ*:

(7) $$P(y|X_{i})=(1-\tau )\delta_{x_{i},y}+\mathrm{\tau P}(y|X_{i}).$$

Here *τ*=0 means the amino acid is fully conserved and *τ*=1 corresponds to the average divergence achieved by matching the context library to the sequence window around the current amino acid. To account for specific terminal branch lengths, first we estimated the average divergence achieved with *τ*=1 when using the K4000 profile library. For this, we combined equations 5 and 6, while only considering the center columns (window size of 1), and averaged over the equilibrium amino acid frequencies *Π*_*c*_:

(8) $$\underset{c\in A}{\sum }\Pi_{c}\overset{K}{\underset{k=1}{\sum }}P(p_{k})p_{k}{\left(0,c\right)}^{2}\approx 0.2$$

Then we can adjust the parameter *τ*for generated profiles to match the expected sequence divergence *δ*according to branch length *d*:

(9) τ=δ/(1−0.2)

The expected sequence divergence $\hat{\delta }$ can be computed either directly from the substitution model or by inverting Kimura's formula [25] (p. 75) for estimating evolutionary distance from sequence divergence:

(10) $$\hat{d}=-\log(1-\delta -0.2\delta^{2})\Rightarrow \hat{\delta }=-\frac{5e^{d}-\sqrt{45e^{2d}-20e^{d}}}{2e^{d}}.$$

## Results and discussion

We evaluated the alignments produced by Mafft [26], Muscle [24], ClustalW [27], Prank [3], POA [14], and variants of ProGraphMSA using the BAliBASE [15] collection of reference alignments and two simulated data sets. Further, the quality of the MSAs is measured by analyzing phylogenies reconstructed from these MSAs [28]. For this we built maximum likelihood and gap phylogeny trees from MSAs of orthologous protein groups with known phylogenetic relations and compared them to reference species trees.

### Command line parameters

Two versions of ProGraphMSA with different evolutionary models were evaluated: 

• ProGraphMSA D based on the indel parameters, stationary amino acid frequencies and Markovian substitution model implemented in Darwin [16,17,21] ( ).

• ProGraphMSA using WAG [22] as substitution model with indel parameters fitted on BAliBASE 3.0.

In general guide trees are built with maximum-likelihood distances (). For non-simulated data sets we further enable context-specific profiles () and empirical amino acid frequencies () as those parameters are intended to aid alignment of real sequence data. For the BAliBASE benchmark and for the simulated data sets we disable special terminal gap probabilities () and forced alignment of M (Methionine) () at the beginning of the sequences. These two parameters are enabled by default to improve the alignment of whole protein sequences. Table 1 summarizes the particular versions and command line parameters used for the other MSA programs.

**Table 1:** Versions and additional options

**Versions and additional command-line parameters**		
POA	poaV2 v1.0.0	-do\_global -do\_progressive blosum80.mat
Prank(+F)	v.111130	+F -twice
Prank	v.111130	-twice
ClustalW	2.0.10	
MUSCLE(-i)	v3.8.31	
MAFFT(-i)	v6.843b	--retree 2 --maxiterate 1000 --globalpair
MUSCLE	v3.8.31	-maxiters 1
MAFFT	v6.843b	--retree 1 --maxiterate 0
ProGraphMSA		--mldist --cs_profile K4000.lib
		(--estimate_aafreqs --end_indel_prob -1 --no_force_align_m)
ProGraphMSA D		--mldist --darwin --cs_profile K4000.lib
		(--estimate_aafreqs --end_indel_prob -1 --no_force_align_m)

Versions and additional parameters of the MSA tools used for comparison with ProGraphMSA.

### BAliBASE 3.0

From the BAliBASE 3.0 benchmark suite we only use the subset of tests that are compatible with the evolutionary models of the tested tools, namely we use the trimmed (BBS*) tests in RV11 (close equidistant sequences), RV12 (more divergent equidistant sequences), RV20 (families with "orphans") and RV30 (divergent subfamilies). All others involve evolutionary events like duplications and rearrangements which are not accounted for in any of the tools in our benchmark and would lead to an arbitrary ranking.

Benchmarking results (Table 2) reveal that among purely progressive alignment methods Muscle (without iterative refinement) and ProGraphMSA perform best. While ProGraphMSA D, which is using the GONNET matrix [21] and an indel model implemented in Darwin [16,17], exhibits a performance similar to ClustalW, the version of ProGraphMSA, optimized on BAliBASE, outperforms it. Mafft-i and Muscle-i, which both perform iterative refinement, outperform all purely progressive methods, while without refinement these tools perform worse or similar to ProGraphMSA.

**Table 2:** Performance comparison on BAliBASE 3.0

**Ranking of MSA tools on BAliBASE**					
	RV11	RV12	RV20	RV30	all
POA	0.26	0.279	0.217	0.183	0.239
Prank+F	0.252	0.6^***^	0.256	0.272^*^	0.357^***^
Prank	0.261	0.607	0.261	0.277	0.363^**^
Mafft	0.245	0.607	0.293^**^	0.321^**^	0.377^**^
ProGraphMSA D (noCS)	0.313^**^	0.63^*^	0.328	0.321	0.41^***^
ProGraphMSA D	0.343	0.647^**^	0.368^**^	0.357^**^	0.44^***^
ClustalW	0.309	0.679^**^	0.338	0.326	0.427
Muscle	0.307	0.663^*^	0.34	0.358^*^	0.428
ProGraphMSA	0.361^*^	0.656	0.383	0.376	0.455
Muscle-i	0.396^**^	0.716^***^	0.358	0.372	0.473^***^
Mafft-i	0.435^**^	0.731	0.446^***^	0.471^***^	0.53^***^
Mummals	0.404	0.766^***^	0.41	0.425^*^	0.514

Displayed are the average true column scores (CS) for the truncated (BBS*) alignments of the RV11, RV12, RV20, and RV30 sets as well as the average over all these sets. Apart from a few exceptions the listing order of the tools implies significantly improving performance. Between each pair of subsequent scores for two different tools we perform a Wilcoxon signed-rank test. Stars indicate a significant difference at a *p*<0.05,*p*<0.01,*p*<0.001 level, respectively. In particular, the use of context-sensitive profiles significantly improves ProGraphMSA D's alignments, whereas our optimized version of ProGraphMSA significantly outperforms ClustalW (*p*=0.0024) but does scarcely not outperform Muscle without refinement (*p*=0.067) at the defined significance level.

### Simulation

For further means of ranking the performance of ProGraphMSA, 10000 protein MSAs with 10 taxa were simulated using ALF [29]. To represent a realistic evolutionary scenario, we chose gamma distributed sequence lengths with a mean length of 300 amino acids and used the WAG model [22] with gamma rate variation among sites. The maximum distance between two sequences was 2 expected substitutions/site. Insertions and deletions were each inserted with a rate of 0.005/substitution and having Zipfian distributed lengths [17] with a mean of 3.5 amino acids and a maximum of 50.

**Figure 3:**
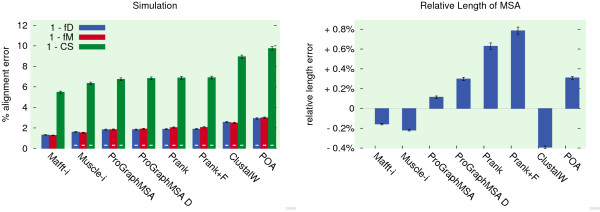
**Quality on simulated data set.** On a simulated data set MSA programs are evaluated by means of three different pair-wise and column-wise alignment error statistics as well as relative error in alignment length. In terms of alignment accuracy on simulated data, ProGraphMSA adopts a position in the midfield after MSA tools with iterative refinement. Surprisingly, Prank has the strongest bias in alignment length, probably due to using p-distances and thus not detecting distant homologies, while ProGraphMSA's results are closest to the reference alignment lengths.

The reconstructed MSAs were compared to the reference alignments by means of relative alignment length, true column score (CS) [15], as well as developer score (fD) and modeler score (fM) [30], which denote the fraction of correctly aligned residue pairs relative to the number of pairs present in the reference MSA (fD) or in the tested MSA (fM), respectively (Figure 3).

Again, Mafft and Muscle produced more precise alignments than either version of ProGraphMSA, but ProGraphMSA outperforms its forefathers POA and Prank. On this simulated data set ProGraphMSA D performs worse than the other variant and in terms of alignment length ProGraphMSA's results are closest to the reference alignments. Surprisingly, Prank significantly over-estimates alignment length, which is also reflected in its fM score. This might be an artefact of errors in the reconstructed guide trees or of Prank not detecting distant homologies due to using p-distances for its guide-tree construction and alignment, and thus underestimating evolutionary distances.

**Figure 4:**
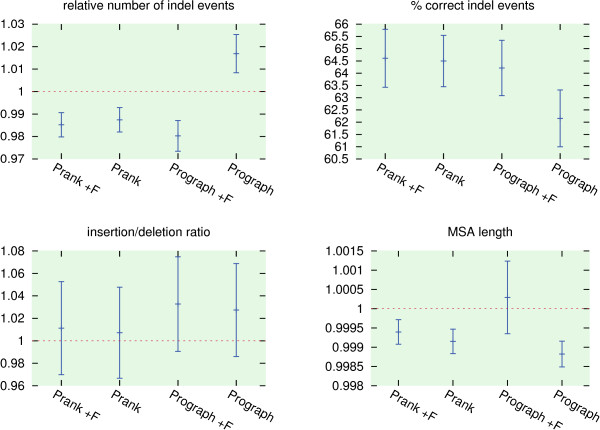
**Gap statistics.** Summary statistics for indel events reconstructed from ancestral sequence alignments produced by four variants of Prank and ProGraphMSA. In particular we evaluate the relative number of indel events, the insertion/deletion ratio, the length of the reconstructed MSAs relative to the reference alignments, and the fraction of correctly inferred indel events. An indel event is defined to be correct, if it starts at the correct column, has the right length, and has been placed on the correct branch of the guide tree.

Further, we simulated a second data set comprising 1000 alignments with known ancestral sequences using the same parameters as before and reconstructed ancestral sequence alignments using Prank and ProGraphMSA. This time the true trees are provided to both tools and they are run with either default parameters or with an option to keep insertions forever ("+F" option in Prank and "-l 0" in ProGraphMSA). The tools are compared using indel statistics similar to those used for evaluating Prank [31] but not relying on a possibly biased reconstruction of indel events by parsimony. Instead, the ancestral sequences inferred by both tools are used to determine the reconstructed indel events (Figure 4).

Overall, both tools exhibit a similar performance in indel reconstruction, with ProGraphMSA+F on average reconstructing alignments with the most accurate length and ProGraphMSA notably reconstructing more indel events than the other tools. The latter can be best explained by ProGraphMSA's feature to revoke erroneous inferences of indel events which appear in the alignment as multiple independent events in the same column leading to a higher error rate.

These combined results indicate that ProGraphMSA is indeed able to compensate errors in the guide tree (Figure 3) while maintaining a comparable precision under ideal conditions, where the true guide tree is provided and gap patterns are congruent with the phylogeny (Figure 4).

### Phylogeny benchmark

The real-data phylogeny reconstruction test [28] uses the precision of phylogenetic tree reconstruction as proxy for MSA quality. The test set consists of more than 10000 groups, each having six sequences sampled from orthologous groups [32] according to established reference topologies of Bacteria, Fungi, and Eukaryota. A MSA program is evaluated by computing an alignment for each of these groups. As indirect quality measure of the alignments, the Robinson-Foulds [33] distance of the reference tree to a PhyML tree reconstructed from the MSAs in question is used.

**Figure 5:**
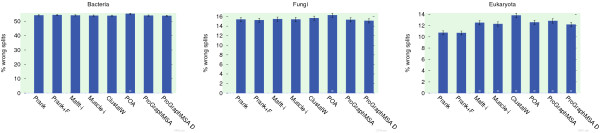
**Tree quality as proxy for MSA quality.** Tree quality as proxy for MSA quality measured as Robinson-Foulds distance (divided by maximum possible distance) of reconstructed PhyML tree to a reference tree. We observe that particularly ProGraphMSA D using the Darwin model is among the best tools in all data sets. A minus sign indicates a significant difference to the best tool using a Wilcoxon signed-rank test. Please note that the vertical axis does not start at zero to highlight the rather mild differences in performance.

In all three data sets (Bacteria, Fungi, Eukaryota) ProGraphMSA D is among the best tools (Figure 5). The Darwin model appears to perform slightly (but not significantly) better than the parameters estimated on BAliBASE. This is probably because BAliBASE's core blocks contain only confident alignments with little uncertain gappy sites. Such training data causes an underestimation of the amount of gaps in the alignment.

**Figure 6:**
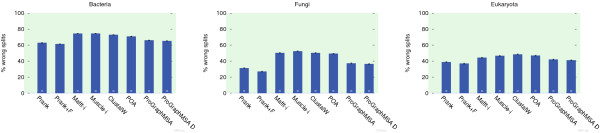
**Tree quality on gap parsimony trees.** Parsimony trees built from only gap information are used to evaluate the quality of MSAs. In the same manner as in the previous figure the relative Robinson-Foulds distance to a reference tree is measured and minus signs indicate a significant difference to Prank, which is always the best tool in this test, followed by ProGraphMSA. This indicates that phylogeny-aware gap placement [3] indeed produces phylogenetically more sensible gap patterns.

In Figure 6 we consider parsimony trees built only on gap information. Prank and ProGraphMSA clearly outperform the other tools (including iterative refinement methods) indicating that phylogeny-aware gap placement [3] actually produces phylogenetically more sensible gap patterns.

**Figure 7:**
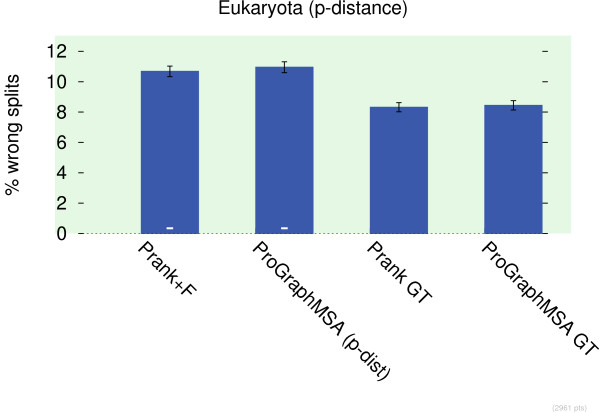
**Quality of guide trees.** Tree quality of guide trees and derived PhyML trees for Prank and ProGraphMSA when using p-distances for guide tree estimation. Probably p-distances bias the guide tree towards the correct topology.

Prank on Eukaryota seems to be a special case as it significantly outperforms all the other tools. Incidentally, on this data set the p-distances used by Prank in conjunction with the NJ algorithm improve the chances of finding the correct topology. A similar effect can be obtained by e.g. taking the square root of ML distances and thus similarly compressing them (results not shown). When using p-distances in ProGraphMSA we achieve a similar precision and we observe that the internal guide trees of both Prank and ProGraphMSA are significantly better than even the reconstructed PhyML trees (Figure 7). Both other data sets favor ML distances.

The authors of the above phylogeny reconstruction test further propose a minimum-duplication test based on larger groups [28]. Here, a MSA tool is considered better, if the reconstructed phylogenies explain the evolution of the leaf sequences with less gene duplications. This test did not yield any significant results and the results have been included in Additional file 1: Figure S1.

### Alternative splicing

**Figure 8:**
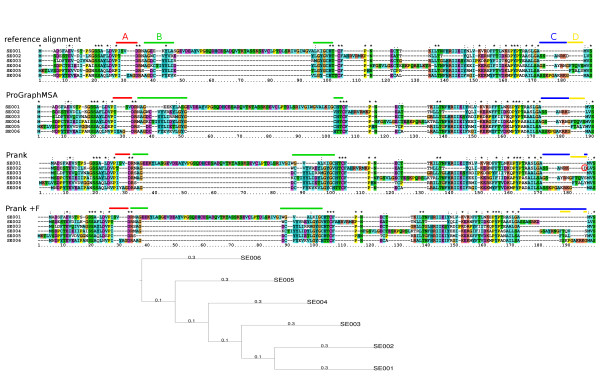
**Example: alternative splicing.** Simulated example of protein sequences along the given phylogeny containing independent insertions at the same site in region A, a long insertion in region B, and alternative splicings in regions C and D. All tested methods have problems with the alignment of the flanking regions (B) of the long insertion and Prank+F fails to align the alternative splicings correctly, as they are not consistent with the phylogeny. Regions C and D are aligned almost correctly by Prank except for the Aspartic acid (red circle) of region C aligned to region D. The independent insertions in region A are detected correctly by ProGraphMSA and Prank+F due to their phylogenetically-aware algorithms.

In a simulation based example (Figure 8) we demonstrate ProGraphMSA's advantages in aligning sequences with alternative splicings and independent insertions at the same sequence position, compared to Prank. Again, both tools were provided with the correct guide trees to exclude guide tree reconstruction as a potential source of alignment errors.

All methods exhibit the usual problems of placing characters at the correct side of long insertions (region B). Due to its heuristic, ProGraphMSA correctly aligned the Methionines (M) at the beginning of the sequence, and the graph-based representation allows for a correct alignment of the alternative splicings (regions C+D) including the insertion inside the alternatively spliced region. Prank+F enforces phylogenetic gap patterns and was thus not able to correctly reconstruct the alternative splicing. Without this feature the regions C and D were aligned almost correctly except for a single Aspartic acid (D) from region C which was aligned with region D. ProGraphMSA aligns this region consistently because it maintains a history of all indel events in its graph structure.

In region A, Prank+F and ProGraphMSA reconstruct the two independent insertions correctly whereas Prank merges these two events. Here it is the penalization of unused graph paths that prevents ProGraphMSA from merging these insertions.

### Execution time comparison

The execution time of ProGraphMSA is dominated by the generation of context-specific profiles. Without this feature the execution time of ProGraphMSA is in the same order of magnitude as the other tools (Table 3). With an increasing number of taxa, we expect distance and tree estimation to consume an increasing share of time due to its quadratic time complexity.

**Table 3:** Average execution times

**Execution time**	
Mafft	27 s
Muscle	34 s
Muscle-i	157 s
ProGraphMSA noCS	191 s
Mafft-i	410 s
ClustalW	435 s
POA	448 s
ProGraphMSA CS	2351 s
Prank	12965 s

Average execution times of the tested MSA tools estimated on the BAliBASE 3.0 benchmark. With a small number of sequences, as in this case, ProGraphMSA spends most of its execution time in the generation of context-specific profiles. With an increasing number of taxa we expect distance and tree estimation to consume an increasing share due to its quadratic time complexity.

In comparison, Prank's performance is dominated by pair-wise alignment and distance estimation for the initial guide tree. We avoid this performance bottleneck by using alignment-free distances [6] for the initial guide tree and compensate for the slightly lower alignment quality by performing an additional iteration of guide tree estimation and progressive alignment.

## Conclusions

ProGraphMSA is a progressive multiple sequence alignment method that combines phylogeny-aware gap placement [3] with a graph-based sequence representation to produce phylogenetically sensible gap patterns while maintaining the flexibility to handle alternative splicings and errors in the guide tree. Our benchmarks reveal that ProGraphMSA presents an unprecedented combination of accuracy on BAliBASE and simulated data, phylogenetically sensible gap patterns, and quality of trees built from the resulting MSAs.

We have successfully applied context-specific profiles [9] to the alignment of multiple sequences. Although the profile generation has only linear time complexity with respect to sequence length, due to the size of the context library the execution time is significantly increased. Nevertheless, we recommend using this feature in ProGraphMSA by default, as context-specific profiles significantly improve alignment quality and the execution time remains competitive in comparison to other tools.

In the future we are planning to implement codon and DNA models and to explore methods of iterative refinement for our alignments. The graph representation allows for adding additional information to the sequences which we intend to adopt for the alignment of proteins with tandem-repeats.

## Availability and requirements

• **Project name:** ProGraphMSA

• **Project home page:**http://www.inf.ethz.ch/personal/sadam/ProGraphMSA

• **Operating system(s):** Platform independent

• **Programming language:** C++

• **Other requirements:** Eigen 3.0, TCLAP 1.1 or higher

• **License:** GNU GPLv3

## Endnote

^a^*δ*_*xy*_=1 if *x*=*y * else 0

## Competing interests

The author declares that he has no competing interests.

## Supplementary Material

Additional file 1 **Figure S1.** Minimum-duplication test. Due to lack of data the minimum-duplication test does not provide a reliable and significant ranking of the tested tools. Click here for file
