# Supplementary figures and images for: Fast and robust multiple sequence alignment with phylogeny-aware gap placement
Source: BMC Bioinformatics. 2012 Jun 13;13:129. doi: 10.1186/1471-2105-13-129 (PMC3495709; doi:10.1186/1471-2105-13-129)

Eukaryota

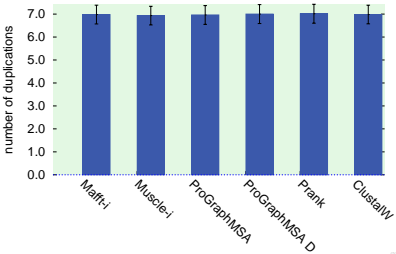

Yeast

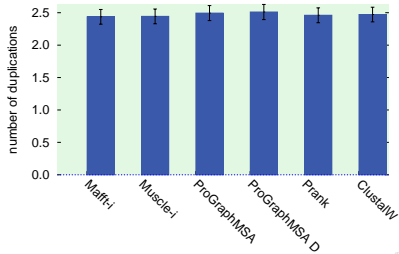

Supplement: Additional file 1 — Figure S1. Minimum-duplication test. Due to lack of data the minimum-duplication test does not provide a reliable and significant ranking of the tested tools. [file 1471-2105-13-129-S1.pdf]
